# Introduction to the special issue on Impact of COVID-19 and cryptocurrencies on the global financial market

**DOI:** 10.1186/s40854-021-00244-2

**Published:** 2021-04-19

**Authors:** Hui Xiao, Xiong Xiong, Weiwei Chen

**Affiliations:** 1 School of Statistics and Research Institute of Big Data, Southwestern University of Finance and Economics, Chengdu, China; 2 College of Management and Economics, Tianjin University, Tianjin, China; 3 Department of Supply Chain Management, Rutgers University, New Brunswick, USA

In the last year, the global outbreak of COVID-19 and the sharply rising price of Bitcoin greatly influence the financial market. Such influences include but are not limited to the trading strategies between different cryptocurrencies, portfolio diversification, foreign exchange markets, and macroeconomic policy. In this 26th issue of Financial Innovation (FIN), Volume 7, No. 2(2021), 22 researchers from 7 countries have used both traditional statistical methods and the newly developed machine learning techniques to analyze how COVID-19 affects the global financial market and how cryptocurrencies affect portfolio diversification. The research results could be beneficial to governments in making macroeconomic policies and financial investors for making better investment decisions.

The first paper, titled “Forecasting and trading cryptocurrencies with machine learning under changing market conditions”, uses machine learning techniques to predict three major cryptocurrencies. The empirical results indicate that the machine learning tools can be used to devise trading strategies for cryptocurrencies.

The second paper, titled “Portfolio diversification benefits of alternative currency investment in Bitcoin and foreign exchange markets”, examines the portfolio diversification benefits of alternative currency trading in Bitcoin and foreign exchange markets. Using the spillover index method, the spillover asymmetry measures, and the frequency connectedness method, this study investigats the dynamics of volatility spillover from Bitcoin to the foreign exchange pairs of major trading currencies. The findings could have significant implications for portfolio diversification and risk minimization.

The third paper, titled “The explosion in cryptocurrencies: a black hole analogy”, uses an analogy between finance and astrophysics to investigate the existence of a mechanism that can describe the explosive increase in the number of traded cryptocurrencies and the cryptocurrency market. The study shows that the changes in the traded cryptocurrencies are a positive function of the number of traded cryptocurrencies in the previous period.

The fourth paper, titled “Regime specific spillover across cryptocurrencies and the role of COVID‑19”, examines the daily return spillover among 18 cryptocurrencies under low and high volatility regimes considering the effect of the COVID-19 outbreak. The analysis reveals that much higher spillovers exist in the high volatility regime during the COVID-19 outbreak.

The fifth paper, titled “Discovering interlinkages between major cryptocurrencies using high‑frequency data: new evidence from COVID‑19 pandemic”, examines the return and volatility spillover between three cryptocurrencies during the pre-COVID-19 period and the COVID-19 period. The findings could be helpful to portfolio managers and policymakers regarding portfolio diversification, hedging, forecasting, and risk management.

The sixth paper, titled “Dynamic connectedness between stock markets in the presence of the COVID‑19 pandemic: does economic policy uncertainty matter?”, investigates the dynamic connectedness between stock indices and the effect of economic policy uncertainty (EPU) in eight countries where COVID-19 was most widespread. The results have important implications for individual investors, portfolio managers, policymakers, investment banks, and central banks.

The seventh paper, titled “The Impact of the COVID-19 Outbreak on Chinese-listed Tourism Stocks”, uses an event study method to analyze the effects of COVID-19 outbreak on stock price movements of China’s tourism industry. The study shows a negative impact exists, and the government’s responses could minimize its impact.

The eighth paper, titled “The macroeconomic effects of COVID‑19 in Montenegro: a Bayesian VARX approach”, examines and assesses appropriate macroeconomic policy responses of the Montenegrin Government to the outbreak of COVID-19. The study shows that the current government’s response is efficient and suggests some useful methods for developing the anti-pandemic and overall development strategy.

